# Gonadal cycle of *Corbicula fluminea* (Bivalvia: Corbiculidae) in Pampean streams (Southern Neotropical Region)

**DOI:** 10.1371/journal.pone.0186850

**Published:** 2017-10-24

**Authors:** Luciana Cao, Cristina Damborenea, Pablo E. Penchaszadeh, Gustavo Darrigran

**Affiliations:** 1 División Zoología Invertebrados, Museo de La Plata FCNyM-UNLP & CONICET, La Plata, Argentina; 2 Laboratorio de Ecosistemas Costeros-Malacología, Museo Argentino de Ciencias Naturales “Bernardino Rivadavia” (CONICET), Buenos Aires, Argentina; Gettysburg College, UNITED STATES

## Abstract

*Corbicula fluminea* is an aggressive invasive species of bivalve that arrived into the Río de la Plata River between the late 60’s and early 70’s, and dispersed widely throughout the Neotropical region, evidencing a great adaptive flexibility to different environmental conditions. This species is a functional hermaphrodite with larval incubation inside the inner demibranch. Despite its widespread distribution, there are no previous studies of complete gonadal histology and reproductive cycle for this species in the Neotropical region. In this study, the reproductive dynamics of *C*. *fluminea* in a temperate region, the Santa Catalina Pampean stream, Argentina, is described. Samples of 20–30 individuals were collected monthly from April 2003-April 2005 and processed using traditional histological techniques. During the two years of this study, seven spawning events were recognized. Three major spawns occurred in spring and summer, and other four minor ones during summer and autumn. Events of oocyte recovery were observed after spawning. A high number of incubating individuals was detected. The results stressed the difficulty of identifying a particular pattern of gamete release and of spawning behaviour in this invasive species, especially when inhabiting an unstable environment.

## Introduction

Ecosystems are increasingly being modified by ongoing human-mediated transoceanic biotic exchange [1] as a consequence of global trade, agriculture, aquaculture, recreational activities and transportation. The Asiatic clam *Corbicula fluminea* (Müller, 1774), is an invasive species that has colonized aquatic ecosystems worldwide [2], in some cases with great ecological and economic impact, acting as an ecosystem engineer [3]. This species native to South-eastern China, Korea and South-eastern Russia [4] has dispersed to the Americas, Africa and Europe [5], encompassing a great diversity of freshwater ecosystems [6]. The Asiatic clam entered into the Neotropical Region through the Río de la Plata River between the late 60’s and early 70’s [7], possibly by the release of living specimens brought as food on-board in vessels [8]; or by the ballast water of transoceanic ships [9]. Thereafter, this species could have been dispersed from the Río de la Plata River to other aquatic ecosystems by vectors such as fishing baits [9] or accidentally imported in sand batches for artificial beaches [10]. Since then, *C*. *fluminea* has been spread widely and invaded the main hydrological basins of this region. In 1988, it was registered for the first time in the Brazilian Amazon Basin (01°54’S–54°39’W) [11], and is currently distributed from the Colorado River (39°01´S-64°01´W) in the northern limit of the Argentinian Patagonia [12] to Venezuela (10°10´S– 63°30´W) [13].

In the Neotropical Region, *Corbicula fluminea* is sympatric with other three non-native species of *Corbicula*: *C*. *largillierti* (Philippi, 1844), *C*. *fluminalis* (Müller, 1774), and *Corbicula* sp. [14]. In some cases, *C*. *fluminea* displaces its congeneric species [9] and frequently becomes dominant within an invaded habitat, acting as an ecosystem engineer [2] and causing physical alterations to these ecosystems [15]. The effect of these modifications on the structure of the benthic macroinvertebrate communities is evident, changing their taxonomic composition and generally having a negative impact over the most common taxa [15]. In addition, when the habitat provides important services such as water supply for drinking, irrigation, or refrigeration in power plants, etc., the invasion by *C*. *fluminea* generates a negative economic impact [14].

The broad dispersion of *Corbicula fluminea* evidences a great adaptive capacity, for instance, being able to develop in both cold and hot climates. Crespo et al. [16] describe *C*. *fluminea* as a freshwater species, which is able to colonize upper estuarine waters with different conditions of salinity and temperature. Undoubtedly, this great invasive potential is also related to its wide spectrum of reproductive strategies. Invasive species display a successful reproductive strategy that allows them to invade new habitats and spread rapidly. Rapid maturity at a small size together with other characteristics of the reproductive cycle of *C*. *fluminea*, are aspects that intend to maximize its reproductive effort. Genetic variability generated by triploidy, hybrid condition, clonal reproduction and the presence of unusual ameiotic breeding systems in *Corbicula*, are mechanisms that also contribute to its success [17].

Previous studies of *Corbicula fluminea* demonstrated that the reproductive tissue fractions (developing or ripe gametes) do not evidence clear cycles [18]. Instead, this species might be capable of responding rapidly throughout the year to suitable environmental conditions by spawning.

The knowledge on the reproductive dynamics of an invasive species is considered a key tool for planning and implementing control strategies [19]. In spite of this, studies that focus on the gonadal cycle of *Corbicula fluminea* are scarce worldwide. Ituarte [20] first studied this subject for *Corbicula* (*C*. *largillierti*) in South America. More recently, Mansur et al. [21] summarized the available information on the reproduction of *C*. *fluminea*, showing controversial results obtained by different authors. This species is generally regarded as hermaphrodite and capable of self-fertilization [22]. However, in Asia low number of dioecious individuals has been reported being related with environmental factors [23]. Kraemer et al. [24] described *C*. *fluminea* as a simultaneous hermaphrodite. Despite this species is commonly regarded as an androgenic [25, 26], Park & Chung [27] registered a case of parthenogenesis without self-fertilization.

As previously referred, gonadal development is well known for other species of *Corbicula*, such as *C*. *largillierti* [20], *C*. *fluminalis* and *C*. *australis* [17], *C*. *japonica* [28,29], and *C*. *leana* [27,30]. However, detailed studies on the gonadal histology and reproductive cycle of *C*. *fluminea* in the Neotropical region have not yet been performed. Since *C*. *fluminea* is an aggressive invasive species that can withstand different climates and environmental conditions, reaching high population densities, the aim of the present study was to describe the reproductive dynamics of *Corbicula fluminea*, establishing its histological gonadal cycle and spawning periods in a population from a Pampean stream (temperate Neotropical Region) and to compare its results with those of previous studies.

## Materials and methods

The study was carried out in Santa Catalina stream, Buenos Aires Province, Argentina (36°53’04.5”S-59°55’25.22”W). Samples were collected monthly from April 2003-April 2005, excluding November 2004. In the field, physical and chemical parameters such as water temperature, dissolved oxygen, conductivity, salinity and TDS were measured. Hydrological parameters such as total rain fall and stream mean flow rate were provided by the Instituto de Hidrología de Llanuras de Azul (IHLLA). The sampling area was delimited by a cylindrical sampler of 0.07 m^2^ area, which was pushed by hand into the sediment up to a depth of 10 cm. The sediment was sieved *in situ* through a mesh (1 mm of pore diameter), and all specimens collected. Sampled specimens of *C*. *fluminea* were returned to the laboratory where they were fixed with Zenker’s solution [31]. The maximum length (size) of each individual was measured with a dial caliper as the greatest linear distance between the anterior and posterior shell margins.

For histological analysis, samples consisted in 20–30 individuals of all sizes found in each month (a total of 692 individuals, 6–30 mm long). The Authority that provide the permission to collect for research is the Dirección de Fauna y Flora, Buenos Aires Province, Argentina. Because *Corbicula fluminea* is an invasive species, there is no restriction on its capture. The field studies did not involve endangered or protected species.

Individuals <6 mm long were excluded from the analysis due to the impossibility of identifying them at species level. After fixation, they were dehydrated and imbedded in Paraplast®. Thereafter, they were cut in 10 μm thickness sections, stained with Mayer’s hematoxylin and eosin and then observed under the microscope. The stages of gonadal development were established following the description for *C*. *japonica* by Rybalkina et al. [29], with introduced modifications in postmature oogenetic stages (Table 1).

**Table 1:** Concordance between the stages of gonadal development used in the present study and those described for *C*. *japonica*.

Rybalkina et al. [29]	Present study	
Oogenic and Spermatogenic stages	Oogenic stage	Spermatogenic stage
Early gametogenesis	Immature	Immature
Active gametogenesis	Premature	Premature
Pre-spawning	Mature	Mature
Spawning	Spawning	Spawning
	Spawned	

Oocyte maximum length was measured in 145 individuals with an optic microscope under 400X magnification. Only those oocytes showing conspicuous nucleoli were considered. Finally, incubated larval periods were determined in the histological sections by the presence of developmental stages and juveniles in the inner demibranch brood chambers [17].

The information on which this work is based comes from the analysis of 1,372 microscopic preparations, deposited in the Colección Malacológica del Museo de La Plata (FCNyM-UNLP), Argentina MLP-MA 14477.

## Results

The physical, chemical and hydrological parameters of the stream during the study period are shown in Table 2. Water temperature exhibited seasonal variations: the maximum value was registered in March 2004 (23.4°C) and the minimum in May 2003 (8.2°C). Mean flow rate range was 0.042 m^3^/sec (March 2004) -1.026 m^3^/sec (December 2003). The lowest values were recorded from January 2004 to June 2004. The lowest values of total rain fall occurred in May 2004 (7.1 mm) and August 2004 (11.8 mm), whereas the highest values occurred in April 2003 (157 mm), November 2003 (169.6 mm) and April 2004 (134.8 mm). The conductivity values varied between 401 and 721 μS during the sampled period.

**Table 2:** Physical, chemical and hydrological parameters in Santa Catalina stream during the sample period. The lack of data is due to technical problems.

Sample	Water temperature (°C)	Mean flow rate (m^3^/sec)	Total rain fall (mm)	TDS (mg/l)	O_2_ (mg/l)	pH	Salinity (‰)
Apr-03	13.8	-	157.0	285	9.26	7.74	1.0
May-03	8.2	-	64.8	307	11.86	7.69	0.0–1.0
Jun-03	11.0	0.378	12.7	-	-	-	1.5
Jul-03	10.2	0.349	91.7	315	13.39	8.08	1.0
Aug-03	10.5	0.765	30.3	364	6.7	7.91	1.05
Sep-03	14.3	0.508	56.2	277	8.8	7.91	2.0
Oct-03	14.0	0.691	68.3	197	5.4	7.66	
Nov-03	16.9	0.295	169.6	-	5.7	7.90	0.0
Dec-03	20.3	1.026	98.4	-	-	-	-
Jan-04	-	0.144	79.8	-	-	-	-
Feb-04	18.6	0.067	12.5	283	3.4	7.40	3.0
Mar-04	23.4	0.042	36.5	274	6.2	7.65	2.5
Apr-04	11.8	0.126	134.8	265	7.5	7.55	3.0
May-04	9.6	0.106	7.1	297	10.2	7.43	2.0
Jun-04	15.2	0.135	36.2	280	8.4	8.60	2.0
Jul-04	9.0	0.478	76.7	264	9.9	8.17	2.0–3.0
Aug-04	13.1	0.698	11.8	-	9.9	7.62	2.0
Sep-04	13.8	0.218	16.7	-	12.9	7.77	2.0
Oct-04	19.7	-	-	312	8.3	7.69	3.0
Dec-04	22.6	-	-	271	7.2	7.52	1.5
Jan-05	20.6	-	-	232	8.1	-	2.5–3.0
Feb-05	23.3	-	-	215	5.1	7.69	1.5
Mar-05	18.1	-	-	310	6.2	8.60	2.0
Apr-05	-	-	-	-	-	-	-

**Fig 1:**
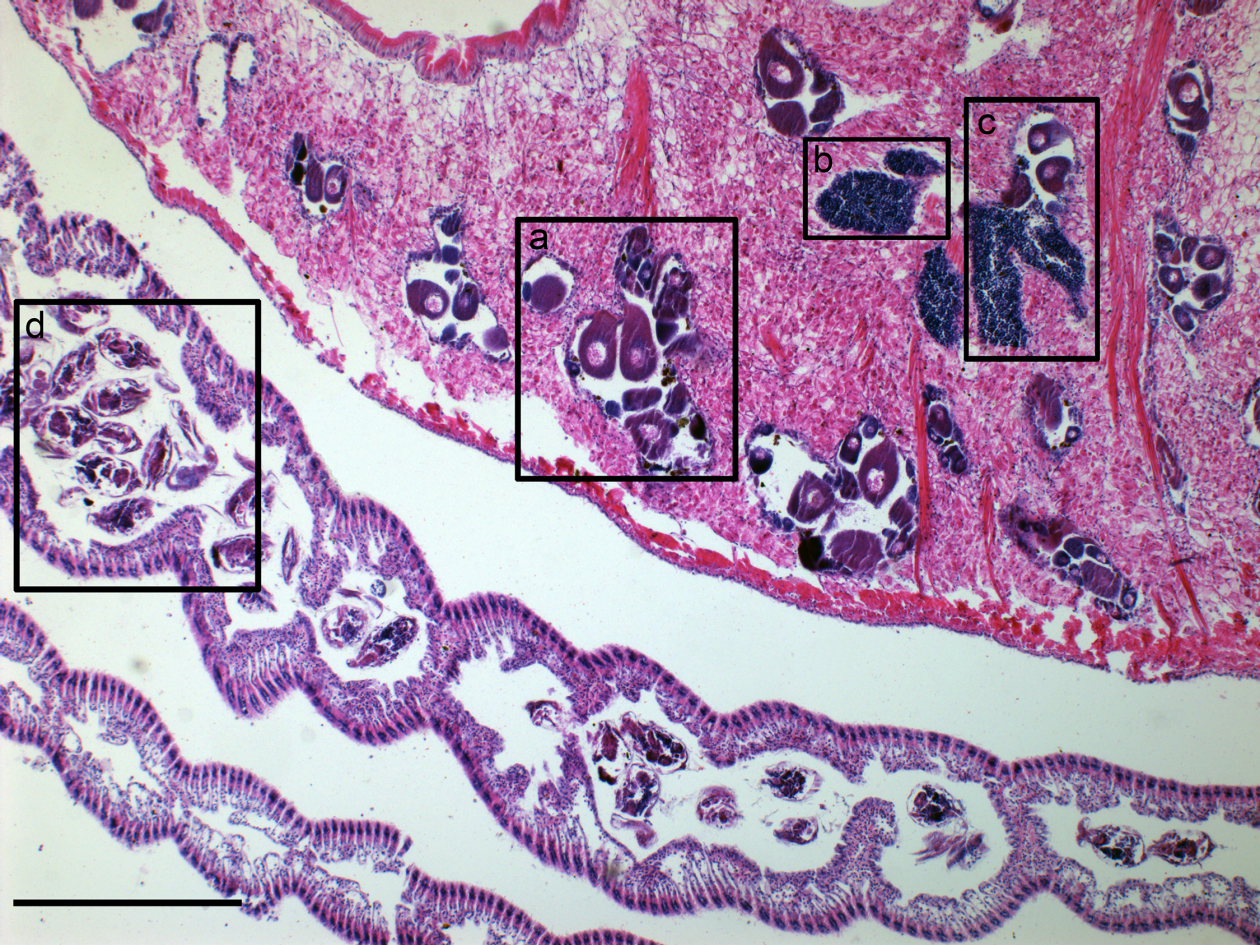
Hermaphroditism in *Corbicula fluminea*. General aspect of the visceral mass showing an oogenic follicle (a), a spermatogenic follicle (b), a mixed follicle (c) and larvae in the inner demibranch (d). Scale bar 200 μm.

The collected individuals ranged from 6–30 mm long, with a mean length of 17.6 mm (Table 3). The percentage of hermaphrodites (with oogenic, spermatogenic and mixed follicles) varied from 60% to 100% of examined specimens in all samples, while most of the remaining individuals were females (only oogenic follicles), as shown in Fig 1. The presence of males (exclusively with spermatogenic follicles) was only registered in October 2004 and January 2005 (2.7% and 4%, respectively). In hermaphroditic specimens, the proportion of oogenic and mixed follicles was usually higher than that observed for spermatogenic follicles, except for a few individuals >16 mm long, in which spermatogenic follicles reached the same or higher proportion than oogenic follicles. It was evidenced that the first size of specimens with oogenic follicles differentiated was smaller (8 mm) than the first size of specimens with spermatogenic follicles (9 mm). There was also a difference in the mean size at first maturity, being smaller for oogenic (11.8 mm) than spermatogenic (14.6 mm) follicles.

**Table 3:** Size and sex data of *Corbicula fluminea* from each sample.

Sample	N	Size (mm)			% F	% M	%H	First size OF (mm)	First size SF (mm)	First size mature oocytes	First size mature sperm	% with FG
		Max	Min	Mean								
Apr-03	26	23	6	14.5	40.0	0	60.0	8	9	15	16	0
May-03	30	24	11	17.5	23.3	0	76.7	11	11	11	14	10.0
Jun-03	32	25	8	16.5	6.2	0	93.7	8	8	11	12	12.5
Jul-03	23	21	8	14.5	0	0	100	8	8	11	14	0
Aug-03	29	22	8	15.0	3.4	0	96.5	8	8	9	9	0
Sep-03	30	21	8	14.5	0	0	100	8	8	8	14	0
Oct-03	30	22	10	16.0	0	0	100	11	12	11	12	3.3
Nov-03	31	23	10	16.5	16.1	0	83.9	10	11	10	12	12.9
Dec-03	25	25	10	17.5	8.0	0	92.0	10	13	10	13	24.0
Jan-04	28	25	14	19.5	3.1	0	96.9	14	14	14	16	0
Feb-04	27	26	11	18.5	28.0	0	72.0	11	12	11	15	6.7
Mar-04	31	24	11	17.5	38.7	0	61.3	11	13	19	19	0
Apr-04	24	27	8	17.5	20.8	0	79.2	8	13	15	16	20.8
May-04	31	25	11	18.0	10.0	0	90.0	11	14	15	17	0
Jun-04	28	27	10	18.5	3.7	0	96.3	10	10	10	22	0
Jul-04	34	21	11	16.0	25.0	0	75.0	11	14	11	-*	2.8
Aug-04	21	27	11	19.0	8.7	0	91.3	11	11	11	16	0
Sep-04	36	28	8	18.0	0	0	100	8	8	13	15	0
Oct-04	47	30	8	19.0	6.8	2.3	90.9	13	13	13	15	4.3
Dec-04	35	21	12	16.5	0	0	100	12	12	12	12	5.7
Jan-05	25	21	8	14.5	8.0	4.0	88.0	8	8	12	18	0
Feb-05	30	25	9	17.5	13.8	0	86.2	9	12	12	14	13.3
Mar-05	30	26	9	17.5	10.0	0	90.0	9	9	9	9	0
Apr-05	35	23	10	16.5	24.3	0	75.7	10	10	12	15	5.4
Mean	-	-	-	17.6	10.7	0.3	88.9	-	-	11.9	14.6	-

*No mature sperm was observed.

N = number of individuals collected; F = female; M = male; H = hermaphrodite; OF = Oogenic Follicles; SF = Spermatogenic Follicles; FG = Follicular Ganglia.

**Fig 2:**
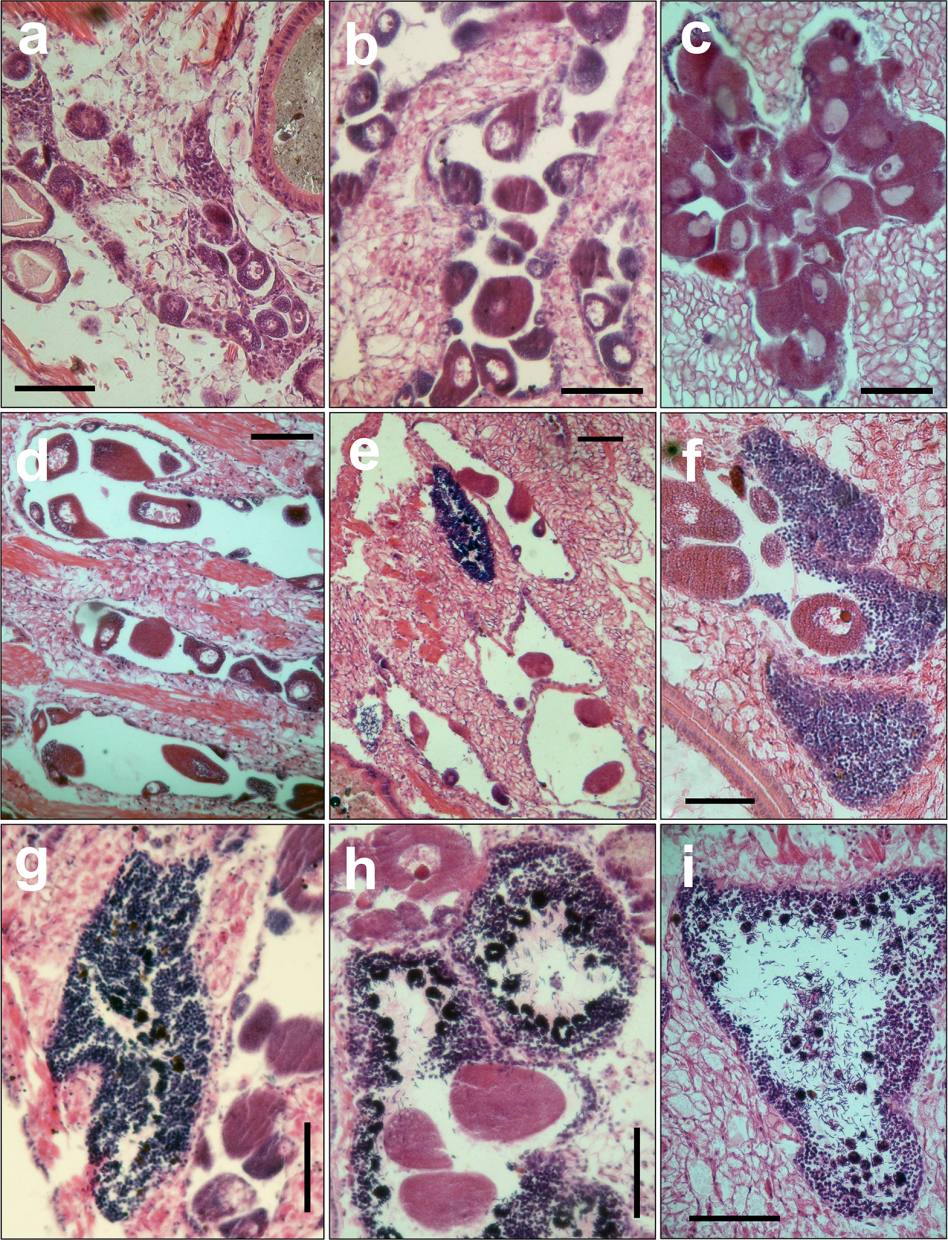
Different stages of gonadal development. Inmature (a), premature (b), mature (c), spawning (d) and spawned (e) oogenic follicles; mixed follicle with inmature spermatogonic stage (f); premature (g), mature (h), and spawning (i) spermatogenic follicles. Scale bars 100 μm.

In accordance with the stages of gonadal development followed in this study, the analysis of the oogenic follicles allowed us to recognize two postmature stages instead of one (Table 1). Hence, during the spawning stage, the follicles were partially empty and there were several mature oocytes in the lumen, some of them elongated due to the spawning process, while during the spawned stage the follicles were almost empty with a few free mature oocytes in the lumen (Fig 2). According to our results, the minimum mature oocyte size from 141 oocytes was 100 μm.

A low proportion of follicular ganglia was observed (0–24%) more commonly associated with spermatogenic follicles. The minimum size of individuals that presented these ganglia was 12 mm, although most of them were >16 mm. In all cases, follicular ganglia were found in spawning or spawned specimens.

**Fig 3:**
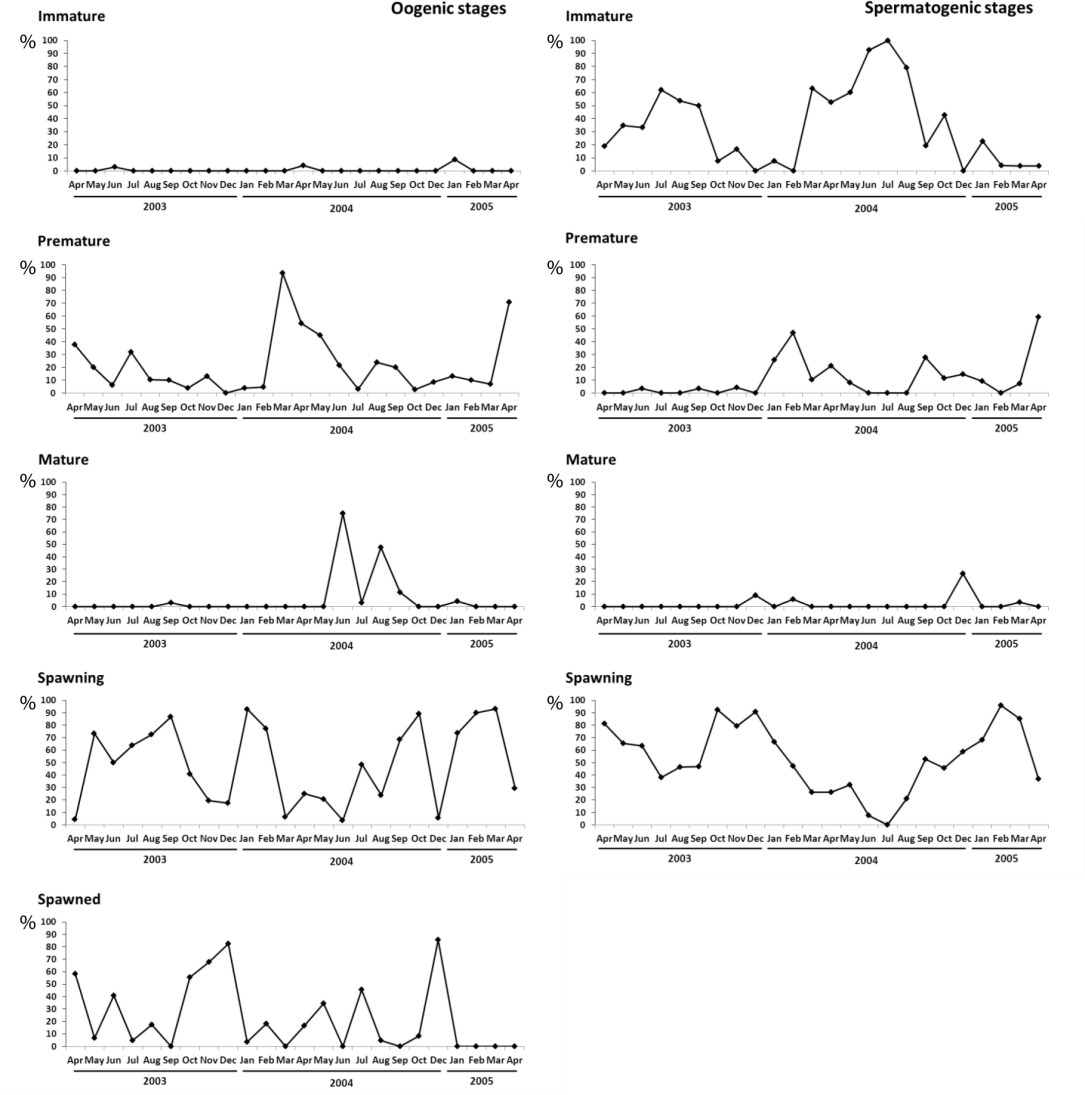
Percentage of individuals at each gonadal stage during the sampled period.

The gonadal development of *Corbicula fluminea* is shown in Fig 3. Neither oogenic nor spermatogenic follicles showed a period of clear inactivity, due to the presence of growing cells, even during spawning events. In the population analyzed, the gonadal development did not appear to present a clear cycle. Immature females were less than 9%. Premature oogenic follicles predominated in March 2004 (93.5%) and April 2005 (70.6%); while mature follicles showed two peaks, in June 2004 (75%) and August 2004 (47.6%). Oocyte major spawning peaks were in May 2003 (73.3%), September 2003 (86.7%), January-February 2004 (92.6% and 77.3%), October 2004 (89.2%) and February-March 2005 (90.0 and 93.1%, respectively). Spawned oogenic follicles were more abundant in December 2003 and 2004 (82.6% and 85.7%, respectively) than in the other sampling dates. Spermatogenic follicles showed immature peaks in July 2003 (61.9%), March 2004 (63.16%) and July 2004 (100%). Mature spermatogenic follicles were <30% with peaks in December 2003 and 2004; while spawning peaks occurred in April 2003 (81.2%), October 2003 (92.3%), December 2003 (90.9%) and February-March 2005 (95.8% and 85.1%). In February-March 2005, a synchrony between male and female spawns was observed.

**Fig 4:**
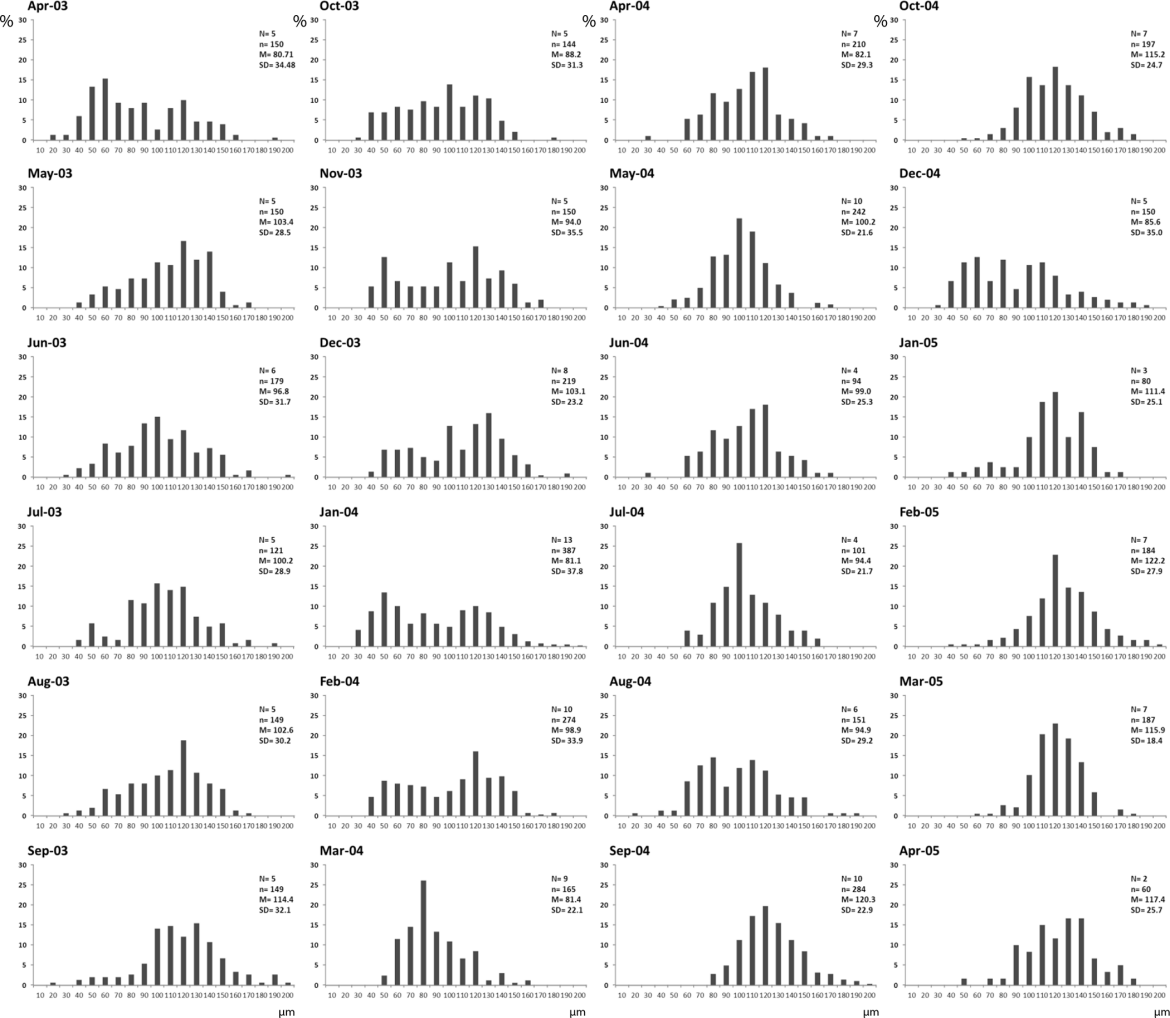
Oocyte size frequencies. N = total number of specimens examined; n = total number of oocytes examined; M = mean oocyte size (μm); SD = standard deviation.

**Fig 5:**
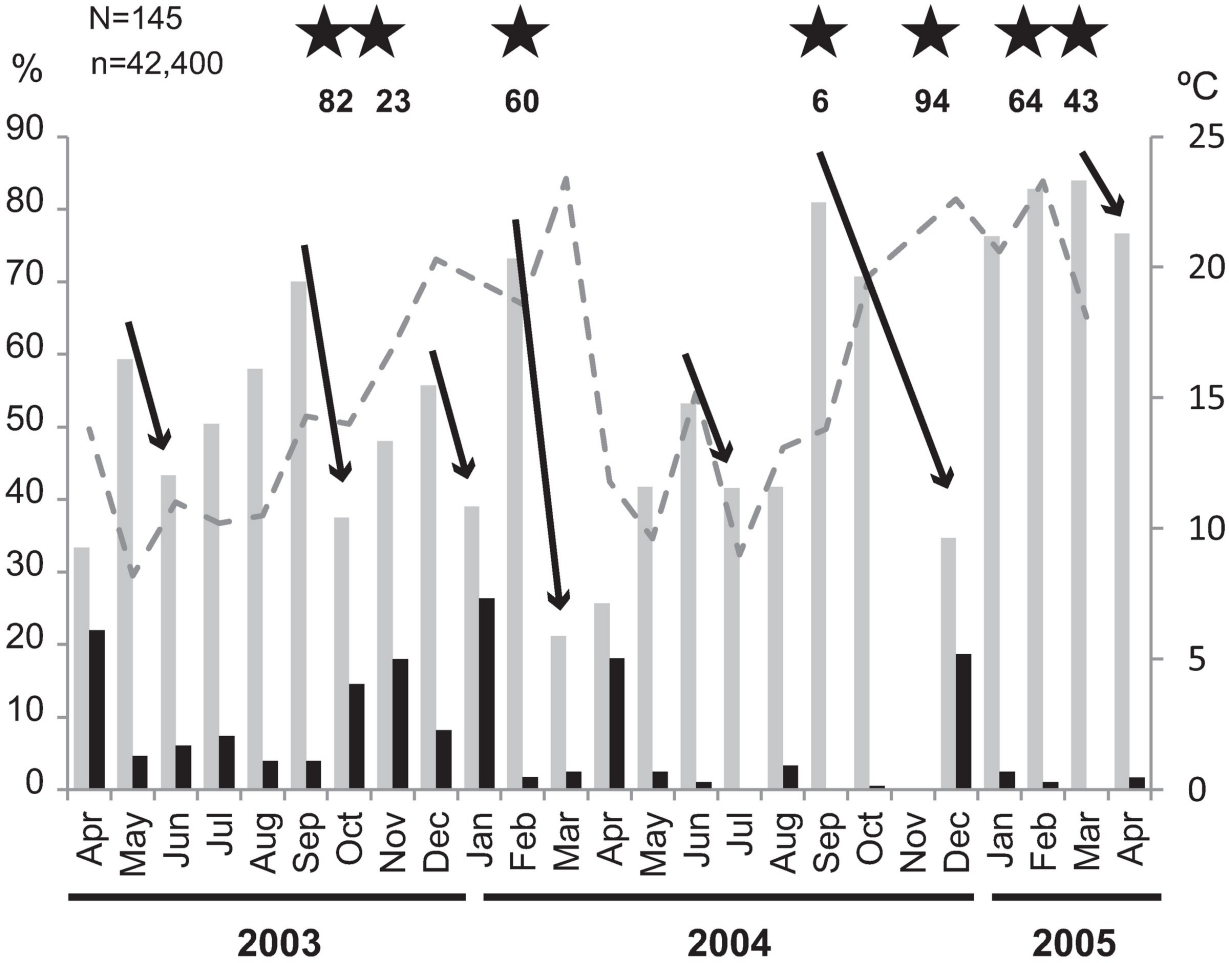
Temporal variation of oocytes of *C*. *fluminea*. Grey bars: oocytes >100 μm; full bars: oocytes <50 μm; dotted grey line: water temperature (right axis); N = total number of specimens considered; n = total number of oocytes considered; arrow: spawning events; star: gill incubation observed and percentages of individuals with larvae.

The analysis of the oocyte size of *Corbicula fluminea* (Figs 4 and 5) evidenced seven spawning events during the study period. Three major spawns (more than 20% of mature oocyte loss) were registered during September-October 2003, February-March 2004 and September-December 2004. Four minor spawns (less than 20% of mature oocyte loss) occurred during May-June 2003, December 2003-January 2004, June-July 2004 and March-April 2005. Major gonadal recovering events occurred in April 2003, November 2003, January 2004, April 2004 and February and March 2005. During these periods, the percentage of growing oocytes was 18–26.4% (Fig 4).

Individuals with incubated larval stages were observed in October and November 2003, February, September and December 2004, and February and March 2005, after spawning events, which is consistent with the results analyzed from gonadal development. Developing larvae are incubated in the inner demibranch brood chambers. The highest percentages of individuals incubating larvae were observed in October 2003 (82.1%) and December 2004 (94.3%) when two major spawns occurred, and in February 2004 (60.0%) (Fig 5). No larval incubation was observed during the spawns of May-June 2003, February-March 2004 and June-July 2004. The temperature measured after these spawning events was always below 15°C (Fig 5).

## Discussion

The lotic system studied in the present work underwent water flow and water level variations during the sampling period (Table 2). Rain fall is the main factor that determines water level and water flow rate in this aquatic system. Rain fall values varied between 169.6 mm/month (November 2003) and 7.1 mm/month (May 2004). Water flow also changed in the same period.

Despite the fact that *Corbicula fluminea* can reach high abundances under local conditions, environmental factors can have major impacts on its population densities and distributions. McMahon [32] indicates that, contrary to what can be expected for an invasive species, *C*. *fluminea* has a relatively low physiological tolerance to changes in abiotic factors, such as temperature, salinity, air exposure, pH, calcium and dissolved oxygen concentrations. The Unionoidea from North America show more physiological resistance than *C*. *fluminea* [32]. Additionally, Modesto et al. [33] predicted that environments that present high temperature values combined with low salinity and low water flow (with low charge of sediment) can support higher densities of *C*. *fluminea*.

While it is well known that *Corbicula fluminea* shows great ability as a freshwater invader, its invasive fitness decreases in brackish waters, even when salinity values are low [34]. According to this, studies established a salinity value of 5 ‰ as top limit of tolerance [22]. Salinity values in Santa Catalina stream did not go over 3 ‰, so this is not a limiting factor for the development of *C*. *fluminea*. This species is also intolerant to moderate hypoxia conditions, being restricted to well oxygenated areas [35] with optimal values between 10.50 mg L^-1^ and 8.30 mg L^-1^ [36]. In Santa Catalina stream, the mean value of dissolved oxygen was 8.50 mg L^-1^, but it fluctuated reaching lower levels in summer. In addition, the thermal preference of *C*. *fluminea* ranges from 2°C to 37°C, and temperatures lower to 2°C cause death of individuals and filtration levels are inhibited below 30°C [22]. In the present study, the lowest temperature registered was 8.2°C while the highest was 23.3°C. *C*. *fluminea* is not well adapted to air exposure conditions, although it is reported to survive means of 26.8 days and 13.9 days in near 100% humidity at 20°C and 30°C, respectively, declining to respective means of 8.3 days and 6.7 days at near 0% humidity [37]. Water level reduction and solar light exposition in a Neotropical aquatic system can disturb the structure in populations of *C*. *fluminea* [38]. Thus, Modesto et al. [33] observed a reduction in the density of *C*. *fluminea* during a drought in Mondego Estuary (Portugal).

The reproductive cycle in bivalves generally involves sexual reproduction, with dioecious or hermaphrodite specimens, external fertilization and a veliger larval stage [39]. However, many groups of bivalves like the species of *Corbicula* show exceptions to this general reproductive behavior (Table 4). Some of them also exhibited parthenogenesis, in which oocytes are activated without fertilization [45], as in case of *Corbicula fluminea* [27].

**Table 4:** Summary of the main biological characteristics of *Corbicula* species. F = female; M = male.

Adapted from [35]	*C*. *fluminea* (= *C*. *manilensis*)	*C*. *largillierti* (Philippi, 1844)	*C*. *leana* Prime, 1867	*C*. *japonica* Prime, 1864	*C*. *australis* Deshayes, 1830	*C*. *fluminea*
Longevity (years)	1 to 4	2.5 to 4		2		
Length at maturity (mm)	6 to 10	10 to 11				11.9 (F); 14.6 (M)
Hermaphroditism	X (self-fertilizing)	X	X	X	X (self-fertilizing)	X
Position of broods	Inner demibranchs (I.D.)	(I.D.)		(I.D.)	(I.D.)	(I.D.)
Fertilization	internal	Internal	internal	internal	internal and external	internal
Fecundity	400–735 (veligers/clam/day)	11,000 (veligers/clam)				
Juvenile size at release (μm)	200–250	225–240			230–240	
Number of annual reproductive events	2–3	2				variable (2–3)
Freshwater	X	X		X	X	X
Brackish water			X			
References	[27,32,35,40–44]	[20,42]	[28]	[17]	[27,30]	this study

The production of free-living larvae is rare in freshwater environments, and occurs in a few bivalve species, such as the invasive *Dreissena polymorpha* and *Limnoperna fortunei* [46]. The physiological mechanisms that allow larvae during the early stages of development to face the intense osmotic stress imposed by freshwater environments still needs to be elucidated [39]. Larvae of *C*. *fluminea* that inhabits the Neotropical region avoid the osmotic stress because fertilization occurs inside the paleal cavity and larvae are incubated inside gill water tubes (or brood chambers). According to Morton [40] and Aldridge and McMahon [41], larvae of *C*. *fluminea* go through trocophore, veliger and pediveliger stages, being released as a D-shaped form with straight hinged shells. In temperate climates, immature individuals are released from gill chambers being almost juvenile, while in subtropical climates, the release occurs at a pediveliger stage [47].

The knowledge on the gonadal development in an invasive bivalve species can be used as a tool for generating control strategies, and consequently applying programs that aim at avoiding their dispersion in natural [48] and artificial [49] ecosystems. Nevertheless, considering that *Corbicula fluminea* is an aggressive invader, studies on its reproductive biology in the Neotropical region are scarce [50].

Hermaphroditism is usually more frequent in freshwater than in marine bivalves [27]. Species of *Corbicula* present different reproductive strategies according to the type of environment they inhabit. In freshwater, they can be hermaphrodite with larval incubation inside gill chambers, while they are usually dioecious, oviparous and non-brooding in estuaries [4,27]. Among freshwater environments, hermaphroditism is more frequent in lentic than in lotic systems [34]. Although *Corbicula fluminea* is indeed a hermaphrodite species, the nature of this hermaphroditic condition is controversial. Several studies described it as a functional [27, 51–53] or protandric hermaphrodite [40]. Morton [23] also described variable reproductive strategies, i.e. capable of being dioecious or hermaphrodite according to environmental conditions. The reproductive tissues observed in individuals from Santa Catalina stream consisted in oogenic, spermatogenic and mixed follicles within the visceral mass, as in the case of the individuals from the United States described by Park and Chung [27]. The proportion of hermaphrodite specimens was remarkably high in all of the samples analyzed. These observations are in agreement with the results obtained by Berry [54] in Malaysia and by Massoli and Callil [55] in Brazil. Our results showed a type of hermaphroditism in *C*. *fluminea*, in which oocytes appeared and maturated before the sperm, and oogenic follicles were the most abundant, as described by Park and Chung [27]. Previous studies in other species of *Corbicula* indicated that oogenic follicles are usually more abundant than the spermatogenic ones [20, 27, 52, 55], being particularly common in individuals that were <20 mm in shell length. These observations are in agreement with the results of the present study, in which the proportion of spermatogenic follicles increases in individuals ≥16 mm in shell length.

The size of mature oocytes differed between species. In our study, the mature oocyte mean size for *C*. *fluminea* was 102 μm, while according to Park & Chung [27] it ranged between 150 and 170 μm. For other species of *Corbicula*, mean mature oocyte size was reported as 110–130 μm in *C*. *leana* and 70 μmin *C*. *japonica* [29].

The number of reproductive events, which means release and fertilization of reproductive cells, is variable in *Corbicula* species in general and particularly in *Corbicula fluminea*. Several authors described two annual reproductive events that took place in spring and summer [40,41,53,56–60], while others reported the presence of a single [43,44,61–63] or of three reproductive events [64]. These differences could be related to divergences in methodological approaches (gonadal cycle studies or presence of larvae). Other potential reasons for reported variation in the reproductive cycle of *C*. *fluminea* could be influences of water temperature [20,28,29,65], phytoplankton abundance [44,64,66], or even variations of metallothionein concentrations between individuals [67]. In the present study, a combination of gonadal spawns and incubating larval periods showed three annual spawning events with presence of incubated larval stages and another three spawns not followed by larval incubation. Despite the most important reproductive events occurred during spring, this population did not show a pattern in the number of reproductive events, unlike reported by other authors [18,27,52,55]. This could be related to the reproductive strategies and features of the life cycle of a typically invasive species, like rapid dispersion, and high densities [19]. In the studied population at Santa Catalina stream, this reproductive behavior could be due to the alternating presence of suitable and unsuitable environmental conditions through time. These conditions were most likely affected by variations in temperature, local rain fall and mean water flow rates (Table 2) resulting in temporal environmental instability.

In our study, *C*. *fluminea* did not exhibit periods of sexual inactivity, as already reported by Massoli and Callil [55], although gonadal resting periods were described for other species of *Corbicula*, such as *C*. *japonica* in the Kievka River, Russia [29]. Considering that both the sampled environments (stream and estuary) and the studied species were different, the presence or absence of resting periods could be due to the fact that the minimum temperatures registered in the estuary of the Kievka River are lower (reaching up to 0°C in January) than those in Santa Catalina stream. As a consequence of low temperatures, the population of *C*. *japonica* might have stopped follicular growth and incubating larval stages. Another reason can be the reproductive plasticity exhibited by *C*. *fluminea*, which allows the continuous renewal of reproductive cells [18].

Park & Chung [27] detected the presence of intrafollicular embryos in *C*. *fluminea*, which in turn confirmed the potentiality of self-fertilization described by Kraemer [52]. In this study, no intrafollicular embryos were found, although the spawning synchrony between sperms and oocytes observed in February-March 2005 might allow the occurrence of this phenomenon.

Kraemer [52] reported for the first time the presence of follicular ganglia in *Corbicula fluminea*. Posteriorly, Ituarte [20] detected the presence of this ganglia specially associated with spermatogenic follicles, in specimens of *C*. *largillierti* from the Rio de la Plata River. More recently, Park & Chung [27] found them in *C*. *fluminea* always associated with mature spermatogenic portions of the reproductive tissue. In this study, although their presence was more common in spermatogenic follicles, they were also found in hermaphroditic and oogenic follicles.

In the present study, larval incubation was only observed in the inner demibranchs of *Corbicula fluminea*, in contrast with the results of Park & Chung [27] and Martins et al. [68], who additionally found a few cases of larval incubation in the outer demibranchs. The condition of incubated larval stages in the outer demibranchs was also reported in specimens of *C*. *possoensis* from Lake Poso, Indonesia [69], despite the modifications in the inner demibranch structure observed during incubation.

In summary, *Corbicula fluminea* is a functional hermaphrodite with larval incubation inside gill chambers. Larval incubation took place when water temperature was higher than 15°C. The first size of specimens with differentiated oogenic follicles was smaller (8 mm) than the first size of specimens with spermatogenic follicles (9 mm). Also, the mean size of first maturity was variable between follicle types, being smaller for oogenic follicles than for spermatogenic follicles.

*Corbicula fluminea* demonstrated a great reproductive plasticity, which is common to most invasive species. This species appears to be able to rapidly respond to appropriate environmental conditions for gamete spawning, having multiple events of spawn throughout the year, particularly in unstable habitats like Santa Catalina stream. This was clearly evidenced by the difficulty in establishing patterns of gamete release as well as of gonadal recovery.
